# Impact of chiglitazar on glycemic control in type 2 diabetic patients with metabolic syndrome and insulin resistance: A pooled data analysis from two phase III trials

**DOI:** 10.1111/1753-0407.13484

**Published:** 2023-10-18

**Authors:** Zhiqiang Ning, Guoqiang Ai, Bo Chen, He Yao, Haixiang Cao, Desi Pan, Xianping Lu

**Affiliations:** ^1^ Shenzhen Chipscreen Biosciences Co., Ltd. Shenzhen China

**Keywords:** chiglitazar (carfloglitazar), insulin resistance, metabolic syndrome, peroxisome proliferator‐activated receptors pan‐agonist, type 2 diabetes

## Abstract

**Background:**

To evaluate the glycemic control effects of vhiglitazar (carfloglitazar), a novel peroxisome proliferator‐activated receptor pan‐agonist, in patients with type 2 diabetes mellitus (T2DM) with metabolic syndrome (MetS) or insulin resistance (IR) using pooled data analysis of two phase III clinical trials.

**Methods:**

Data were collected from two randomized phase III clinical trials in China, comparing chiglitazar to placebo or sitagliptin in T2DM patients. The MetS was defined by the Adult Treatment Panel III MetS criteria, and IR was defined by homeostatic model assessment for insulin resistance (HOMA‐IR) ≥4.31 (male) or 4.51 (female). The main end point of this analysis was glycemic control in the different arms within each subgroup.

**Results:**

In the MetS subgroup, changes in glycated hemoglobin (HbA1c) from baseline at week 24 in the chiglitazar 32 mg, chiglitazar 48 mg, and sitagliptin 100 mg arms were −1.44%, −1.68%, and −1.37%, respectively; *p* < .05 was obtained when chiglitazar 48 mg was compared with sitagliptin. In the IR subgroup, the changes in HbA1c were −1.58%, −1.56%, and −1.26% in chiglitazar 32 mg, chiglitazar 48 mg, and sitagliptin 100 mg arms, respectively; *p* < .05 was obtained when chiglitazar 32 mg was compared with sitaligptin. The two doses of chiglitazar demonstrated a greater reduction in fasting plasma glucose and 2 h postprandial plasma glucose than sitagliptin in the pooled population and in the MetS and IR subgroups.

**Conclusions:**

Chiglitazar shows promising efficacy for glycemic control in patients with T2DM associated with MetS or IR. Further prospective trials are required to validate these findings.

## Graphical abstract

**Highlights**

- Risk for atherosclerotic disease increases when type 2 diabetes mellitus (T2DM) is associated with metabolic syndrome (MetS).
- T2DM patients who present with MetS or insulin resistance show better improvement with the chiglitazar treatment. These results indicate a preferable population for chiglitazar treatment.

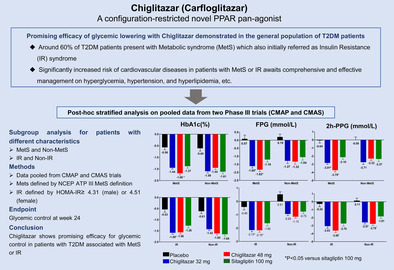

## 1 INTRODUCTION

Type 2 diabetes mellitus (T2DM) is well known as a chronic progressive disease. Despite the existing variant drug classes in clinics, patients eventually fail to respond to most oral antidiabetic drugs and rely on insulin. The key issue is that T2DM patients express long‐standing insulin resistance (IR) and accompanying metabolic syndrome (MetS).

The concept and meaning of MetS have been constantly evolving. In 1998, the World Health Organization recommended the use of the term “metabolic syndrome.” The National Cholesterol Education Program Adult Treatment Panel III (ATP III) devised a definition for MetS in 2001, and the American Heart Association and the National Heart, Lung, and Blood Institute updated the definition in 2005. In the updated definition, MetS is diagnosed if three or more of the following criteria are met: waist circumference ≥102 cm (men) or 88 cm (women), systolic blood pressure ≥130 mm Hg or diastolic blood pressure ≥85 mm Hg or on antihypertensive drug treatment for hypertension, fasting triglyceride (TG) level ≥1.7 mmol/L or on drug treatment for elevated TG, fasting high‐density lipoprotein cholesterol (HDL‐C) level <1.03 mmol/L (men) or 1.3 mmol/L (women) or on drug treatment for reduced HDL‐C, fasting plasma glucose (FPG) ≥5.6 mmol/L or on drug treatment for elevated glucose.^1^

Most studies have shown that MetS is associated with an approximate doubling of cardiovascular disease risk and that the risk for incident T2DM is more than five times higher in individuals with MetS compared with those without the syndrome.^2^ Once T2DM compounds MetS, the risk of atherosclerotic cardiovascular disease (ASCVD) events continues to increase.^3^ Therefore, effective glycemic control in these patients is beneficial for reducing ASCVD.

Peroxisome proliferator‐activated receptors (PPARs) belong to nuclear hormone receptors family, encompassing three isotypes of PPARα, PPARδ, and PPARγ. These isotypes act as key regulators of lipid and glucose metabolism, playing a central role in maintaining energy homeostasis and modulating inflammatory response.^4^ The thiazolidinedione class of PPARγ agonists, which includes rosiglitazone and pioglitazone, has demonstrated significant and enduring effects of glycemic control in patients with type 2 diabetes, acting as a remarkable insulin sensitizer. However, their clinical applications have been restricted due to undesired adverse effects such as weight gain, edema, and congestive heart failure. Due to their distinct and even contrasting roles in proposed activities, it is highly possible to optimize the metabolic actions and safety profiles in patients with type 2 diabetes, especially those accompanied by MetS via targeting all three isotypes.^5^

Chiglitazar is designed with a configuration‐restricted non‐thiazolidinedione chemical structure and acts as a moderately potent and well‐balanced PPAR pan‐agonist, exhibiting unique molecular mechanisms of action in vitro and in vivo,^6^, ^7^ and has been proven to have a good safety profile and promising effects on both glycemic control and lipid modulation in patients with T2DM in China in previous clinical trials.^8^, ^9^ Here, we performed a pooled data analysis of two randomized phase III clinical trials conducted in China to evaluate the glycemic control of chiglitazar in patients with MetS and IR.

## 2 METHODS

### 2.1 Study population and design

This analysis included data from two randomized clinical phase III trials conducted in China, which have been described previously.^8^, ^9^ Both trials enrolled T2DM patients with insufficient glycemic control on strict diet and exercise alone. Patients were treated with chiglitazar 32 or 48 mg for 24 weeks for efficacy and safety evaluations, which were compared with placebo (CMAP study) or sitagliptin 100 mg (CMAS study). Both trials were approved by the ethical committees of all the study centers and were performed in accordance with the ethical standards of the institutional and/or national research committee and with the Declaration of Helsinki and its later amendments or comparable ethical standards. All participants signed written informed consent. A total of 1274 patients were enrolled in these two trials under the same inclusion and exclusion criteria and were subjected to this pooled analysis. The main elements of the trials are summarized in Table 1.

**TABLE 1:** Trials included in the pooled analysis.

Trial No.	CMAP	CMAS
Clinical trial number	NCT02121717	NCT02173457
Treatment arms and patients	Placebo (*N* = 202); chiglitazar 32 mg (*N* = 167); chiglitazar 48 mg (*N* = 165).c	Sitagliptin 100 mg (*N* = 248); chiglitazar 32 mg (*N* = 246); chiglitazar 48 mg (*N* = 245).
Treatment duration	After 24‐week treatment, patients in chiglitazar groups remained on the planned treatment, whereas the placebo group was randomly assigned to receive chiglitazar 32 or 48 mg up to 52 weeks.	24 weeks
Included patients	Type 2 diabetes patients with no oral or injected antidiabetic treatment for at least 12 weeks before screening, aged 18–70 years, body mass index range of 18.5–35.0 kg/m^2^, had insufficient glycemic control (HbA1c ≥7.5% and ≤10.0%) despite a strict diet and exercise regimen.	
Excluded criteria	Key exclusion criteria were type 1 diabetes or type 2 diabetes treated with fibrates or other drug therapies that could alter blood glucose levels during the 12 weeks before randomization; alanine aminotransferase or aspartate aminotransferase level >2.5 times the upper limit of normal levels, estimated glomerular filtration rate <60 mL/min per 1.73 m^2^.	
Primary efficacy end points	Level change in HbA1c at week 24 from baseline.	
Secondary efficacy end points	The proportion of patients meeting HbA1c treatment goal of <7.0% at week 24; the proportion of patients having HbA1c lowered by 0.5% at week 24; the change from baseline over time of fasting plasma glucose, 2 h postprandial plasma glucose, fasting plasma insulin, free fatty acids, homoeostasis model assessment for insulin resistance (HOMA‐IR) and beta‐cell function (HOMA‐B), triglycerides, total cholesterol, high‐density lipoprotein cholesterol, and low‐density lipoprotein cholesterol levels.	

Abbreviation: HbA1c, glycated hemoglobin.

### 2.2 Pooled data analysis

The main objective of this pooled data analysis was to assess the glycemic control of chiglitazar in subgroups of patients who presented with MetS defined by the ATP III MetS criteria and IR defined by the homeostatic model assessment for insulin resistance (HOMA‐IR) ≥4.31 (male) or 4.51 (female) at baseline.^1^, ^10^ Glycated hemoglobin (HbA1c), FPG, and 2 h postprandial plasma glucose (2 h‐PPG) levels were used as glycemic control parameters in the MetS, non‐MetS, IR, and non‐IR subgroups. Changes in HbA1c, FPG, and 2 h‐PPG levels from baseline to week 24 were assessed for all treatment arms within each subgroup.

### 2.3 Statistical analyzes

Treatment arms were compared for changes from baseline in HbA1c, FPG, and 2 h‐PPG at week 24 using a mixed effects analysis of covariance model for repeated measures in all patients and all subgroups. All data, including those collected after discontinuation of the study treatment and use of rescue treatment, were included. The observed change from baseline at each scheduled post‐baseline visit was the dependent variable. The model included the baseline value as a covariate with fixed factors for the baseline HbA1c category, visits, baseline‐by‐visit, and treatment‐by‐visit interactions. Least squares mean (LSM) differences between arms (each chiglitazar dose vs. sitagliptin) and 95% confidence intervals (CIs) were estimated, a value of *p* < .05 was considered statistically significant.

## 3 RESULTS

### 3.1 Baseline characteristics and overall glycemic control effects

**FIGURE 1:**
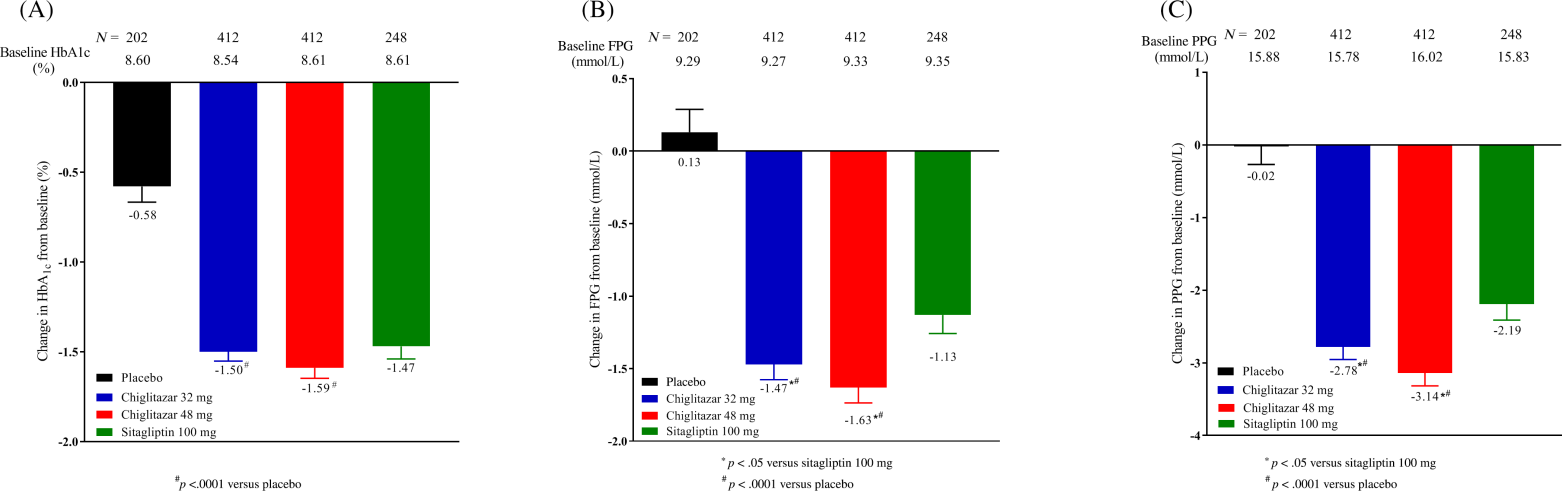
Glycemic control in all patients. *p < .05, refer to comparison of the change at week 24 from baseline in HbA1c (A), FPG (B), and PPG (C) between chiglitazar and sitagliptin. ^#^ *p* < .0001, refer to comparison of the change at week 24 from baseline in HbA1c, FPG, and PPG between chiglitazar and placebo. FPG, fasting plasma glucose; HbA1c, glycated hemoglobin; PPG, postprandial plasma glucose. [Correction added on 28 October 2023, after first online publication: in figure 1B, ‘Baseline PPG’ has been corrected to ‘Baseline FPG’ while in figure 3C, ‘Baseline FPG’ has been corrected to ‘Baseline PPG’.]

A total of 1274 T2DM patients from two phase III trials were pooled for this analysis, including 202 in the placebo arm, 248 in the sitagliptin 100 mg arm, 412 in the chiglitazar 32 mg arm, and 412 in the chiglitazar 48 mg arm. Demographic and baseline characteristics were generally comparable between the chiglitazar and control groups (Table 2). By the 24th week of treatment, chiglitazar at both doses showed significant glycemic control compared with the placebo in terms of HbA1c, FBG, and 2 h‐PPG. Treatment with chiglitazar 48 mg also resulted in greater reduction in FPG and PPG levels than treatment with sitagliptin 100 mg (Figure 1).

**TABLE 2:** Demographic and baseline characteristics in the pooled population.

	Placebo (*N* = 202)	Chiglitazar 32 mg (*N* = 412)	Chiglitazar 48 mg (*N* = 412)	Sitagliptin 100 mg (*N* = 248)
Sex, male (%)	124 (61.4)	252 (61.2)	260 (63.1)	160 (64.5)
Age (years), mean (SD)	51.2 (10.0)	51.1 (9.6)	51.2 (9.7)	50.7 (9.5)
Diabetes duration, years, mean (SD)	1.4 (2.5)	1.4 (2.3)	1.4 (2.3)	1.4 (2.7)
BMI, mean (SD)	26.1 (3.0)	25.9 (3.1)	26.0 (3.2)	25.8 (3.2)
HbA1c_,_ %, mean (SD)	8.6 (0.7)	8.5 (0.7)	8.6 (0.7)	8.6 (0.7)
HbA1c ≥8.5% at screening, *n* (%)	103 (51.0)	210 (51.0)	215 (52.2)	129 (52.0)
FBG, mmol/L, mean (SD)	9.3 (2.3)	9.3 (2.1)	9.3 (2.2)	9.4 (2.2)
HDL‐C, mmol/L, mean (SD)	1.2 (0.3)	1.1 (0.3)	1.1 (0.3)	1.2 (0.3)
2 h‐PPG (mmol/L), mean (SD)	15.9 (3.8)	15.8 (3.5)	16.0 (3.5)	15.8 (3.8)
TG, mmol/L, mean (SD)	2.0 (1.3)	2.2 (1.7)	2.1 (1.9)	2.1 (1.5)
Free fatty acids, mmol/L, mean (SD)	0.5 (0.2)	0.5 (0.2)	0.5 (0.2)	0.5 (0.2)
HOMA‐IR, mean (SD)	5.1 (3.9)	5.1 (3.9)	5.3 (4.6)	4.7 (3.3)
HOMA‐β, mean (SD)	48.8 (35.7)	49.9 (39.5)	56.6 (170.5)	43.9 (34.9)

Abbreviations: 2 h‐PPG, 2‐hour‐postprandial glucose; BMI, body mass index; FBG, fasting blood glucose; HbA1c, glycated hemoglobin; HDL‐C, high‐density lipoprotein cholesterol; HOMA‐IR, homeostatic model assessment for insulin resistance; TG, triglyceride.

### 3.2 Glycemic control in MetS subgroup

**FIGURE 2:**
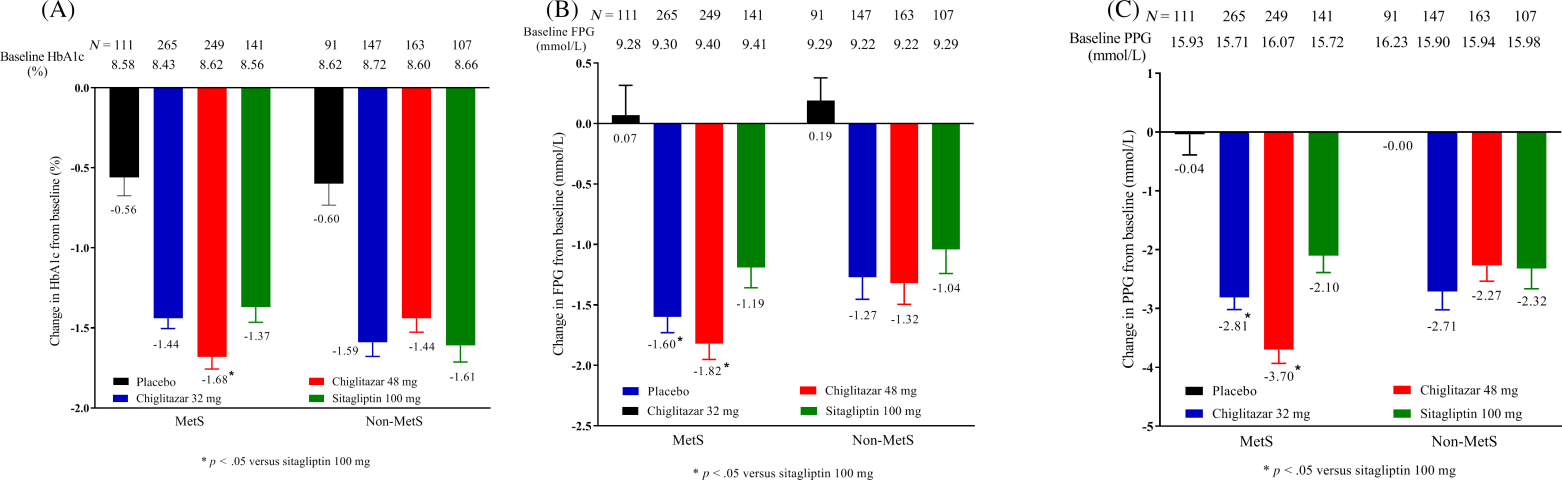
Glycemic control in MetS and non‐MetS patients. *p < .05, refer to comparison of the change at week 24 from baseline in HbA1c (A), FPG (B), and PPG (C) between chiglitazar and sitagliptin within each subgroup (MetS or Non‐MetS). FPG, fasting plasma glucose; HbA1c, glycated hemoglobin; MetS, metabolic syndrome; PPG, postprandial plasma glucose.

Among the 1274 patients, 766 met three or more criteria of the ATP III MetS definition and were assigned to the MetS subgroup, and the other 508 patients were assigned to the non‐MetS subgroup. At baseline, there were 111, 265, 249, and 141 patients in the placebo, chiglitazar 32 mg, chiglitazar 48 mg, and sitagliptin 100 mg arms of the MetS subgroup. After 24 weeks of treatment, the numbers of patients reverting to non‐MetS were 20 (18%), 98 (37%), 100 (40%), and 53 (38%) in the respective arms. At week 24, the HbA1c levels in the chiglitazar 48 mg and chiglitazar 32 mg arms decreased from baseline by 1.68% and 1.44%, respectively. A statistically significant distinction was observed when comparing the LSM difference of chiglitazar 48 mg with those of sitagliptin 100 mg, yielding a *p* value of .0175 (Figure 2A). In the MetS subgroup, both chiglitazar doses significantly decreased FPG and 2 h‐PPG levels compared with sitagliptin (Figure 2B,C).

### 3.3 Glycemic control in IR subgroup

**FIGURE 3:**
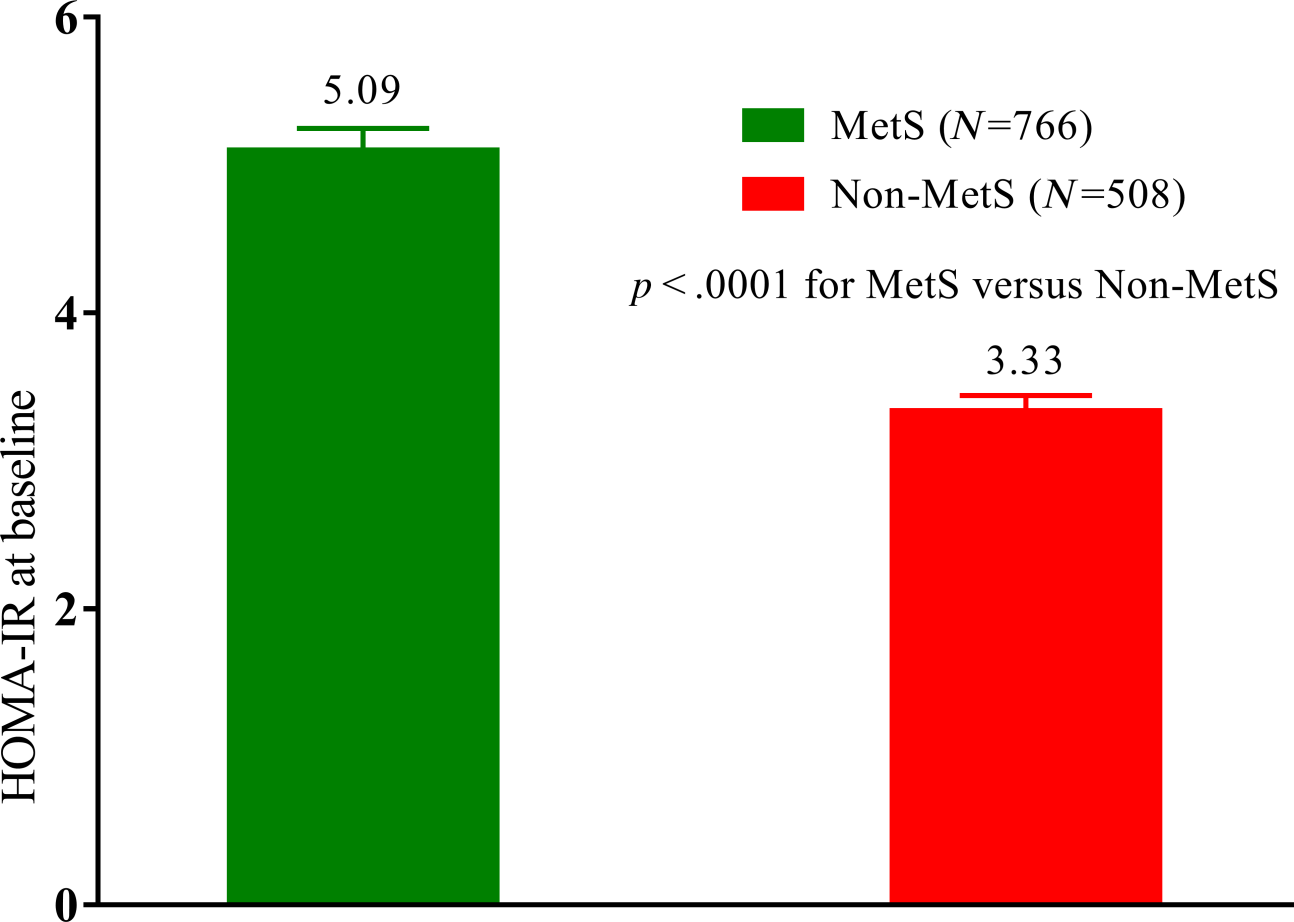
Baseline HOMA‐IR in MetS and non‐MetS patients. *p* < .0001, refer to comparison of the mean of HOMA‐IR at baseline between MetS and non‐MetS patients. HOMA‐IR, homeostatic model assessment for insulin resistance; MetS, metabolic syndrome.

**FIGURE 4:**
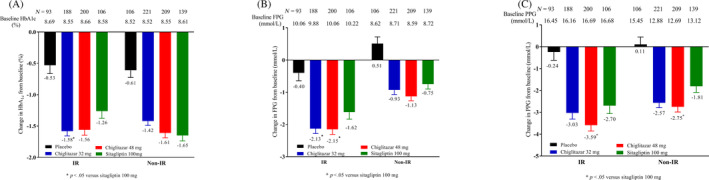
Glycemic control in IR and non‐IR patients. *p < .05, refer to comparison of the change at week 24 from baseline in HbA1c (A), FPG (B), and PPG (C) between chiglitazar and sitagliptin within each subgroup (IR or non‐IR). FPG, fasting plasma glucose; HbA1c, glycated hemoglobin; IR, insulin resistance; MetS, metabolic syndrome; PPG, postprandial plasma glucose.

In addition, HOMA‐IR was higher in patients with MetS than in those without (Figure 3). HOMA‐IR is a surrogate index of IR that indicates an inappropriate state of cellular response to insulin hormones present in both T2DM and MetS. This may be related to the preferential hypoglycemic efficacy of chiglitazar in patients with MetS. According to previously defined criteria in the Chinese population, patients can be divided into IR and non‐IR subgroups. In IR subgroup patients, changes in HbA1c at week 24 from baseline were −0.53%, −1.58%, −1.56%, and −1.26% for the placebo, chiglitazar 32 mg, chiglitazar 48 mg, and sitagliptin 100 mg arms, respectively (*p* = .0225 for chiglitazar 32 mg vs. sitagliptin 100 mg, and *p* = .0532 for chiglitazar 48 mg vs. sitagliptin 100 mg). Chiglitazar 48 mg significantly reduced FPG and PPG levels compared to sitagliptin 100 mg, whereas chiglitazar 32 mg significantly reduced FPG levels compared to sitagliptin 100 mg (Figure 4).

## 4 DISCUSSION

In this pooled data analysis, chiglitazar showed more favorable glycemic control in patients with IR. This is consistent with the proposed mechanism of action of chiglitazar in insulin sensitization. Our investigation, aligning with APT III findings, substantiates the intrinsic link between MetS and IR.^3^ The pooled data analysis affirmed this connection, revealing a HOMA‐IR value of 5.09 for the MetS subgroup in contrast to 3.30 for the non‐MetS subgroup. ATP III also indicated that IR is at the root of MetS,^1^ thus, rectifying IR assumes paramount significance in the therapeutic approach to MetS. Given the heightened sensitivity of 2 h‐PPG as an indicator of IR in comparison to FPG, it reinforces the discernment that addressing 2 h‐PPG offers a more refined lens through which to gauge and manage IR.^11^ Both chiglitazar doses exerted a greater reduction of 2 h‐PPG compared to sitagliptin 100 mg within the MetS and IR subgroups. This trend aligns seamlessly with prior research highlighting the inverse correlation between IR and the glycemic response to dipeptidyl peptidase‐4 inhibitors.^12^ Furthermore, this observation resonates with the superior efficacy of PPARγ agonist pioglitazone over sulfonylureas and metformin in patients with a distinct subset of diabetes characterized by severe insulin‐resistant diabetes.^13^

Although the frequency of classic cardiovascular risk factors such as elevated levels of low‐density lipoprotein cholesterol and blood pressure are similar in populations with or without T2DM, the unique pathological characteristics of IR and hyperglycemia lead to increased risk of cardiovascular disease in T2DM.^14^ Various mechanisms have been proposed to explain how elevated plasma glucose levels promote atherosclerosis, in brief, nonenzymatic glycosylation of proteins and lipids induced by hyperglycemia could promote oxidative stress and proinflammatory response in multiple type of cells within diabetic vasculature. Chronic inflammation could induce IR, whereas a meta‐analysis with data from 11 prospective studies suggests that IR is independently associated with an increased risk of hypertension in the general population.^15^ Together with accompanying dyslipidemia of MetS, it may accelerate the pathological progress of vascular damage to atherosclerosis in patients with T2DM and particularly accompanying with MetS.^3^ Regarding the proved and durable efficacy in glycemic‐lowering and potent insulin‐sensitizing effect of PPARγ agonists, coupled with lipid‐modulation and anti‐inflammatory effects by individual PPAR isotypes, the PPAR pan‐agonist may have compounded cardiologic benefits.^16^, ^17^, ^18^

In summary, chiglitazar demonstrated efficacy in managing glycemia and modulating lipid profiles among patients with type 2 diabetes. Notably, its effectiveness was more pronounced within subgroups characterized by IR and concomitant MetS. It could be a potential new clinical option to treat patients with T2DM, as well as bring potential benefits for other metabolic disorders and reduce the risk of ASCVD owing to its polypharmacological targeting of multiple mutual risk factors. The sample size used in this subpopulation analysis was limited, and the analysis results need to be further verified in corresponding larger‐scale populations.

## DISCLOSURE

The authors are employees of Shenzhen Chipscreen Biosciences, Co., Ltd., the developer and holder of chiglitazar. The authors declare no conflict of interest.
